# MiR-10a-5p-Mediated Syndecan 1 Suppression Restricts Porcine Hemagglutinating Encephalomyelitis Virus Replication

**DOI:** 10.3389/fmicb.2020.00105

**Published:** 2020-02-20

**Authors:** Shiyu Hu, Zi Li, Yungang Lan, Jiyu Guan, Kui Zhao, Dianfeng Chu, Gencheng Fan, Yuguang Guo, Feng Gao, Wenqi He

**Affiliations:** ^1^Key Laboratory of Zoonosis Research, Ministry of Education, College of Veterinary Medicine, Jilin University, Changchun, China; ^2^State Key Laboratory of Genetically Engineered Veterinary Vaccines, Yebio Bioengineering Co., Ltd. of Qingdao, Qingdao, China

**Keywords:** porcine hemagglutinating encephalomyelitis virus, miR-10a-5p, Syndecan 1, virus replication, antiviral mechanism

## Abstract

Porcine hemagglutinating encephalomyelitis virus (PHEV) is a single-stranded RNA coronavirus that causes nervous dysfunction in the infected hosts and leads to widespread alterations in the host transcriptome by modulating specific microRNA (miRNA) levels. MiRNAs contribute to RNA virus pathogenesis by promoting antiviral immune response, enhancing viral replication, or altering miRNA-mediated host gene regulation. Thus, exploration of the virus–miRNA interactions occurring in PHEV-infected host may lead to the identification of novel mechanisms combating the virus life cycle or pathogenesis. Here, we discovered that the expression of miR-10a-5p was constitutively up-regulated by PHEV in both the N2a cells *in vitro* and mice brain *in vivo*. Treatment with miR-10a-5p mimics allowed miR-10a-5p enrichment and resulted in a significant restriction in PHEV replication, suggesting widespread negative regulation of the RNA virus infection by miR-10a-5p. The outcomes were also evidenced by miR-10a-5p inhibitor over-expression. Luciferase reporter, quantitative real-time PCR (qRT-PCR), and western blotting analysis further showed that Syndecan 1 (SDC1), a cell surface proteoglycan associated with host defense mechanisms, acts as a target gene of miR-10a-5p during PHEV infection. Naturally, siRNA-mediated knockdown of SDC1 leads to a reduction in viral replication, implying that SDC1 expression is likely a favorable condition for viral replication. Together, the findings demonstrated that the abundant miR-10a-5p leads to downstream suppression of SDC1, and it functions as an antiviral mechanism in the PHEV-induced disease, providing a potential strategy for the prevention and treatment of PHEV infection in the future work.

## Introduction

Porcine hemagglutinating encephalomyelitis virus (PHEV) is a neurotropic coronavirus and causes central nervous system (CNS) disorders and digestive illnesses in piglets (Li et al., 2016). PHEV is an enveloped positive strand RNA virus and coding five structural proteins, including small envelope (E), membrane (M), nucleocapsid (N), spike (S), and hemagglutinin-esterase protein (HE) (Dong et al., 2014). The main host of PHEV infection is the piglets under 3 weeks of age, manifested by vomiting and wasting disease (VWD) or encephalomyelitis, with a mortality rate of nearly 100% (Vijgen et al., 2006). Previous studies have shown that the disease is widespread worldwide (Gao et al., 2011), although it is often in a subclinical state in conventional swine farms owing to the colostrum antibodies and age-related resistance (Rho et al., 2011). By now, there are no effective preventive vaccines or drugs for prevention; thus, research on PHEV infection is vital to prevent outbreaks.

MicroRNAs (miRNAs) are a group of endogenous non-coding RNA and act as post-transcriptional regulators of target genes mRNAs (Panoutsopoulou et al., 2018). The mature miRNAs are single-stranded RNA of ∼20 nucleotides, which participate in almost biological processes, including host–pathogen interactions (Huang et al., 2017). Increasing studies demonstrate that miRNAs are involved in host–virus interactions and actively regulate the replication of pathogenic viruses (Haasnoot and Berkhout, 2011). For example, miR-30e^∗^ inhibits the dengue virus (DENV) replication by restoring IFN-β production (Zhu et al., 2014). Host cells utilized miR-26a to defend against porcine reproductive and respiratory syndrome virus (PRRSV) infection by activating innate antiviral immunity (Jia et al., 2015). MiR-185-5p inhibits hepatitis B virus (HBV) transcription and replication by targeting ETS transcription factor 1 (ELK1) (Fan et al., 2018), whereas miR-520a inhibited HBV replication by inactivating the AKT signaling pathway (Sun et al., 2018). Furthermore, several viruses hijack host miRNAs to promote their replication, such as miR-146a, which promotes HBV replication by targeting zinc finger e-box binding homeobox 2 (ZEB2) (Wang and Li, 2018). Our previous research found that some miRNAs, such as miR-21a and miR-142-5p, are involved in PHEV infection (Li et al., 2017; Lv et al., 2017), and these findings lead to a better understanding of miRNA-mediated regulation of PHEV infection.

In this paper, we found that the cellular miR-10a-5p was up-regulated by PHEV infection and the enriched miR-10a-5p conversely repressed PHEV replication. Further studies suggested that the miR-10a-5p may potentially bind to the 3′-UTR of SDC1 mRNA and may play an antiviral effect on PHEV-infected cells. The data marked that the altered miR-10a-5p directly affects viral pathogenesis and acts as a potential inhibitor of PHEV replication, thereby implying a new therapeutic strategy for PHEV-infected disease. Therefore, understanding the role of cellular miRNAs during viral infection may lead to the identification of novel mechanisms to block RNA virus replication or cell-specific regulation of viral vector targeting.

## Materials and Methods

### Cells and Viruses

The human embryonic kidney cells (HEK293T) and mouse neuroblastoma N2a (N2a) cells were maintained in Dulbecco modified Eagle medium (DMEM; Sigma) supplemented with 10% fetal bovine serum (Gibco, United States) and 1% penicillin/streptomycin. These cells were incubated at 37°C in a humidified atmosphere with 5% CO_2_. The PHEV strain HEV 67N (GenBank accession: AY048917) was propagated and titrated on N2a cells.

### Animals

BALB/c mice were provided from the Laboratory Animal Center of Jilin University. All animal studies were conducted according to experimental practices and standards approved by the Animal Welfare and Research Ethics Committee of the College of Veterinary Medicine, Jilin University, China (no. KT201904002).

### Antibodies

Mouse monoclonal antibody against PHEV was generated in our laboratory. Mouse monoclonal anti-beta actin was purchased from Proteintech, and rabbit polyclonal anti-SDC1 was obtained from Abcam. Horseradish peroxidase secondary anti-mouse and rabbit IgG antibodies were obtained from Proteintech. Goat anti-mouse IgG–Alexa 568 and goat anti-rabbit IgG–Alexa 488 were purchased from Cell Signaling Technology.

### MicroRNA Target Prediction

The potential targets of miR-10a-5p were predicted by some bioinformatics website, including DIANA-microT-CDS^1^, miRanda^2^, PicTar^3^, and TargetScan^4^ databases. The target sites of miR-10a-5p in the 3′-UTR region of the potential genes were predicted by TargetScan.

#### *In vivo* and *in vitro* Infection

Three-week-old BALB/c mice were nasally inoculated with 2 × 10^6^ 50% tissue culture infection dose (TCID_50_)/ml of PHEV. The brain tissues were collected on 0, 3, and 5 days post infection. N2a cells were seeded in six-well plates, cultured to 70–80% confluence, and then infected with or without the PHEV at 2 × 10^6^ TCID_50_/ml within the indicated time. Total RNA and protein were extracted and detected by quantitative real-time PCR (qRT-PCR) and western blotting (*n* = 3).

### MicroRNA Array

Treated mice were anesthetized with 3.5% chloral hydrate (1.0 ml/100 g; Sigma), and then cerebral cortex tissue was exfoliated under aseptic conditions. Total RNA was harvested using TRIzol (Invitrogen) according to manufacturer’s instructions. After having passed RNA quantity measurement, the samples were labeled and hybridized on the miRCURY LNA Array (v.18.0). The heat map diagram shows the result of the two-way hierarchical clustering of miRNAs and samples. T indicates PHEV treated group, whereas C represents control group. The result of hierarchical clustering shows distinguishable miRNA expression profiling among samples.

### Plasmid Construction

To construct the wild-type reporter plasmid, the 3′-UTR sequence of mouse SDC1 gene, which contains the putative miR-10a-5p binding site, was amplified and cloned into pmirGLO luciferase reporter vector (SDC1-WT). On the other hand, the sequence of SDC1 mRNA 3′-UTR that contains the mutant binding site was amplified by overlap extension of PCR and then constructed as a mutant-type reporter (SDC1-MUT). All plasmids were verified by DNA sequencing. The primers were designed as follows: SDC1-WT sense primer, 5′-GAG CTC ACC TCG CTT CCC TAA TCT AC-3′; SDC1-WT anti-sense primer, 5′-CTC GAG ACA GGC TCT TCC AAT GTC AC-3′; SDC1-MUT primer 1, 5′-GAG CTC ACC TCG CTT CCC TAA TCT AC-3′, SDC1-MUT primer 2, 5′-CTC ATG CGT ACA ATG CGG TAT GGA CTA TC-3′; SDC1-MUT primer 3, 5′-GAT AGT CCA TAC CGC ATT GTA CGC ATG AG-3′; and SDC1-MUT primer 4, 5′-CTC GAG ACA GGC TCT TCC AAT GTC AC-3′.

#### Transfection and Viral Infection

For siRNA transfection, cells are seeded on a six-well plate to reach 30–40% confluence after 12 h. Then SDC1 siRNA or the negative control siRNA (NC) was transfected with X-tremeGENE HD DNA Transfection Reagent (Roche, Sweden) in OPTI-MEM medium (Gibco), according to the instruction of the manufacturer. For miRNA transfection, cells were plated as the previous method and transfected with miR-10a-5p mimic, miR-10a-5p inhibitor, and negative control (RiboBio). In the viral infection experiment, cells were infected with PHEV at 2 × 10^6^ TCID_50_/ml at 48 h after transfection and were then collected at 24, 48, and 72 h post infection. All the transfection experiments were repeated at least three times.

### Quantitative Real-Time PCR

To analyze SDC1 mRNA expression, total RNA was extracted using TRIzol (Invitrogen) and reverse transcribed into cDNA by using the reverse transcription kit (Takara, Japan). For miRNA isolation and analysis, miRNA was extracted by a miRNApure Mini Kit (cwbio, China), and then, the Bulge-Loop miRNA qRT-PCR Primer Set (RiboBio, China) was utilized to analyze mature miRNA expression. qRT-PCR was performed using SYBR Green Master Mix kit with the StepOne Real-Time PCR System. The relative expression of mRNAs and miRNAs was normalized using GAPDH and U6, respectively, followed by being analyzed by the 2^–Δ^ ^Δ^ ^*Ct*^ method. U6 and mmu-miR-10a-5p primers were purchased from RiboBio. The primers for SDC1 and GAPDH were designed as follows: SDC1 sense primer, 5′-CCT CAT CTT TGC TGT GTG CC-3′; SDC1 anti-sense primer, 5′-GCT TGG TGG GTT TCT GGT AG-3′; GAPDH sense primer, 5′-CTC AAC TAC ATG GTC TAC ATG TTC-3′; GAPDH anti-sense primer, and 5′-ATT TGA TGT TAG TGG GGT CTC GCT C-3′.

### Luciferase Reporter Assay

For the SDC1 mRNA 3′-UTR luciferase reporter assay, HEK293T cells were plated in 12-well plates and co-transfected with 1 μg of SDC1-WT or 1 μg of SDC1-MUT plasmid as described above, along with miR-10a-5p mimics, miR-10a-5p inhibitors, or negative controls (RiboBio). After 48 h post-transfection, the luciferase reporter assay system (Promega) was used to detect firefly and Renilla luciferase activities. These data are represented as the firefly luciferase activity relative to the Renilla luciferase activity. The experiment was repeated at least three times.

### Western Blotting Analysis

The cells and brain tissues were lysed by using Radio Immunoprecipitation Assay Lysis Buffer containing phenylmethylsulfonyl fluoride protease inhibitor (Beyotime). After being lyse on ice for 30 min, the BCA Protein Assay Kit (Pierce) was used to determine protein concentration. The samples were separated on 12% sodium dodecyl sulfate polyacrylamide gel electrophoresis gels and were then transferred to 0.45 μm polyvinylidene fluoride membranes. Non-specific binding was blocked using 5% non-fat dry milk in 0.01 M of phosphate-buffered saline (PBS) for 1 h at room temperature. Following that, membranes were washed five times with PBS and incubated in rabbit polyclonal anti-SDC1 or mouse monoclonal anti-beta actin antibody dilution buffer with gentle agitation overnight at 4°C. Then the membranes were washed and incubated with horseradish peroxidase-linked secondary anti-mouse or anti-rabbit IgG antibodies for 1 h at room temperature. Finally, the binds were detected using ECL reagents (Proteintech).

### Indirect Immunofluorescence Assay

For immunofluorescence staining, N2a cells plated in 24-well plates were infected with PHEV at 2 × 10^6^ TCID_50_/ml. At 48 h post infection, N2a cells were washed twice with PBS and fixed with 4% paraformaldehyde for 15 min and then incubated with 5% non-fat dry milk in PBS for 1 h at 37°C, followed by incubation with a monoclonal antibody against the PHEV (1:1,000) and a polyclonal antibody against SDC1 for 1 h at 37°C. Cells were washed five times with PBS and incubated with goat anti-mouse IgG–Alexa 568 conjugates and goat anti-rabbit IgG–Alexa 488 (1:300) for 1 h at 37°C. After being washed five times with PBS, the nuclei were visualized with 4′,6-diamidino-2-phenylindole (DAPI, 1:500). The stained cells were observed using a confocal microscope.

### Cell Proliferation Assays

Cell growth and viability were measured using Cell Counting Kit-8 (CCK-8) assay kit (Meilunbio, China). The treated cells were grown in 96-well plate with ∼5 × 10^3^ per well and incubated for 24, 48, and 72 h. At the indicated time, 10 μl of CCK-8 solution was added to the cell culture medium for 1-h incubation. The absorbance of the converted dye at 450 nm was measured by a microplate reader.

### Statistical Analysis

All experiments presented were repeated three times. The data are presented the mean ± SD. Statistical analysis was performed with either Student’s *t*-test or one-way ANOVA in GraphPad Prism version 5 software. All data were considered statistically significant at a *p*-value < 0.05.

## Results

### Porcine Hemagglutinating Encephalomyelitis Virus Infection Up-Regulates MiR-10a-5p Expression

**FIGURE 1:**
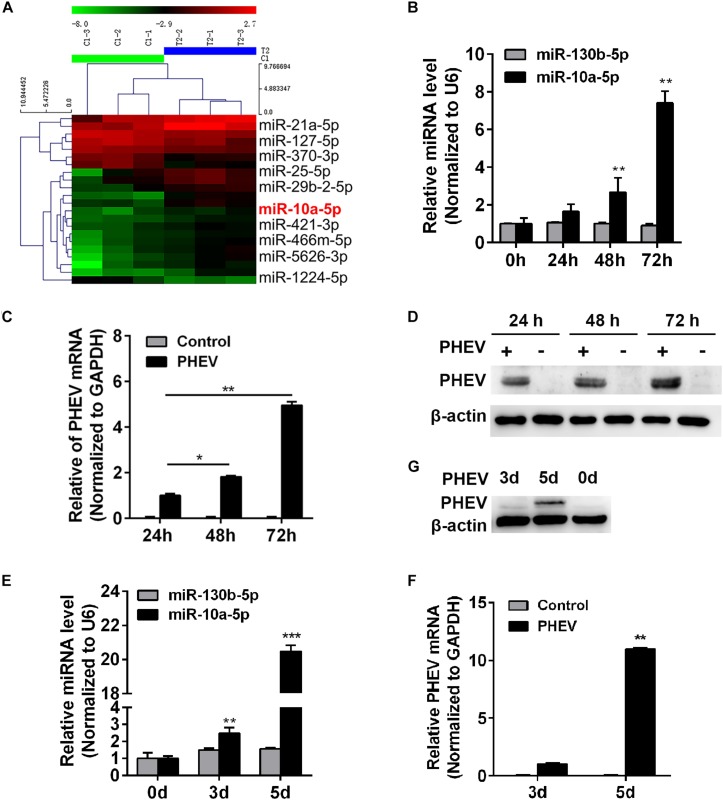
Porcine hemagglutinating encephalomyelitis virus (PHEV) infection up-regulates miR-10a-5p expression. **(A)** MiRNA array of PHEV-infected and uninfected mice. Hierarchical clustering shows distinguishable miRNA expression profiling among samples. **(B)** N2a cells were infected with PHEV at 2 × 10^6^ TCID_50_/ml and harvested at 0, 24, 48, and 72 h post infection. The expression of miR-10a-5p was determined by qRT-PCR. U6 was chosen as a housekeeping gene to normalize miR-142a-3p expression. Another irrelative miR-130b-5p is used as a negative control. **(C)** Cells were treated as described in panel **(B)**, then the total RNA was extracted, and the relative expression of PHEV mRNA was determined by qRT-PCR. Data were normalized to GAPDH. **(D)** N2a cells treated as described in panel **(B)**, and the cell lysates were collected and subjected to examine the expression of PHEV protein by western blotting. Beta actin expression was verified as loading control. **(E)** BALB/c mice were infected with PHEV for 3 or 5 days, and then the cerebral cortexes were harvested, and miR-10a-5p level was determined by qRT-PCR. *N* = 4 mice per group, three independent experiments. **(F)** The lysis from cerebral cortexes of mice as described in panel **(E)** was collected, and then the relative expression of PHEV mRNA was determined by qRT-PCR. **(G)** The cerebral cortical lysis was detected by western blotting, and the PHEV protein was normalized to beta actin. All of data are representative of at least three independent experiments, with the error bars representing the standard deviations (SDs). *p < 0.05, **p < 0.01 vs. normal controls.

Investigating the miRNA profiling during PHEV infection may provide insight into the CNS injury diseases. A miRNA array was previously performed to identify the miRNAs involved in neurological dysfunction as a result of PHEV infection (Figure 1A and Supplementary Data Sheet S1). Of these up-regulated miRNAs, we found that miR-10a-5p was of research value and practical significance. To investigate the role of miR-10a-5p during PHEV infection, N2a cells and BALB/c mice were infected with PHEV. Cell lyses were collected at the indicated time, and the expression of miR-10a-5p RNA and PHEV genome was detected by qRT-PCR (Figures 1B,C), whereas the viral protein levels were determined by western blotting (Figure 1D). The results showed that the miR-10a-5p level was significantly higher than that in the control group at 48 and 72 h post infection, during which PHEV RNA replication and protein expression were remarkably strengthening (Figures 1B–D). Likewise, 3-week-old BALB/c mice were intranasal inoculated with PHEV, and the cerebral cortex was obtained at 0, 3, and 5 days post infection. And we confirmed a heightened expression of miR-10a-5p by qRT-PCR analyses, which is tightly correlative to enhanced PHEV propagation (Figures 1E–G). These data indicate that miR-10a-5p level was up-regulated by the increasing amount of PHEV during infection.

### MiR-10a-5p Suppresses the Replication of Porcine Hemagglutinating Encephalomyelitis Virus

**FIGURE 2:**
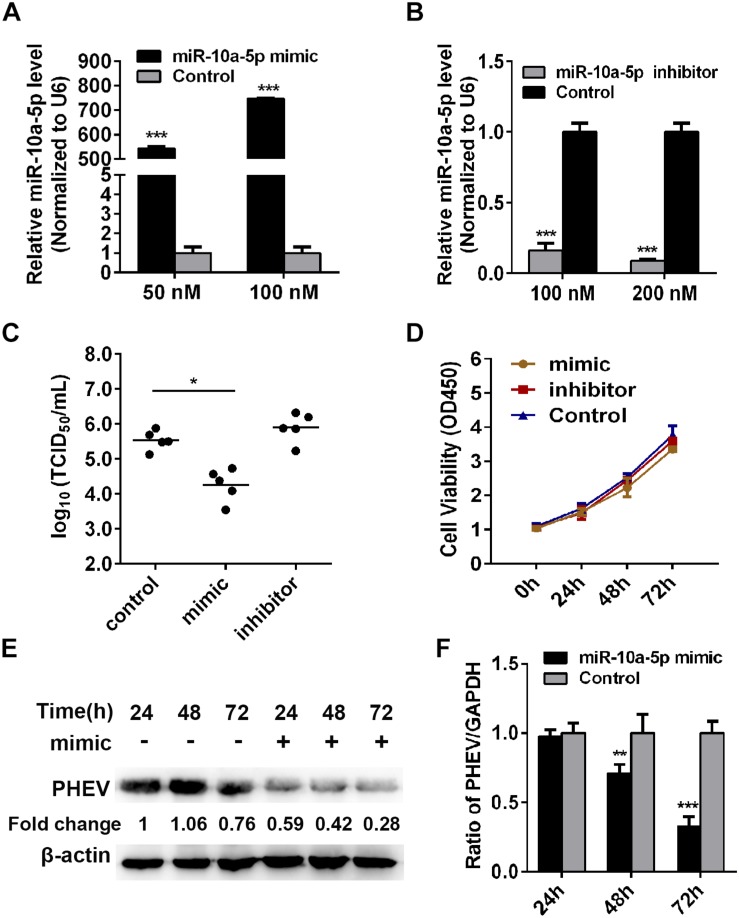
Abundant miR-10a-5p suppresses the replication of porcine hemagglutinating encephalomyelitis virus (PHEV). **(A)** N2a cells were transfected with different concentrations of miR-10a-5p mimic for 24 h, and then the expression of miR-10a-5p was analyzed by qRT-PCR and normalized to U6. **(B)** N2a cells were transfected with 100 or 200 nM miR-10a-5p inhibitor, and miR-10a-5p level was detected by qRT-PCR. **(C)** N2a cells pre-transfected with miR-10a-5p mimic, miR-10a-5p inhibitor, or miRNA NC were seeded in 96-well plates followed by PHEV infection. The cytopathic effect was monitored for 24 to 96 h. The virus titers were determined by TCID_50_/ml and calculated according to the Reed–Muench method. **(D)** Cell Counting Kit- (CCK-8) assay was performed to determine the cell proliferation at the indicated times. **(E)** N2a cells were pre-transfected with 100 nM of miR-10a-5p mimic and then infected with PHEV at 2 × 10^6^ TCID_50_/ml. The cells lystate were harvested at 24, 48, and 72 h post infection, and PHEV protein levels were determined by western blotting and normalized to beta actin. **(F)** Cells were treated as described in panel **(E)** and determined by qRT-PCR by analyzing PHEV mRNA level. GAPDH was chosen as a housekeeping gene for normalization. Data are shown as means ± SD of at least three independent experiments. *p < 0.05; ***p < 0.01.

To examine whether miR-10a-5p has a biological function on PHEV replication, the N2a cells were transfected with miR-10a-5p mimic or inhibitor for 24 h, and then the expression of miR-10a-5p was detected by qRT-PCR. Our results showed a significant increase in the miR-10a-5p level with the mimic transfection at a dose of 50 or 100 nM (Figure 2A). In contrast, the level of miR-10a-5p decreased after treatment with miR-10a-5p inhibitors (Figure 2B). These miR-10a-5p mimic- or inhibitor-treated cells were then seeded in 96-well plates, and virus titers were determined by TCID_50_/ml. As shown in Figure 2C, the RNA virus titers in the miR-10a-5p mimic-overexpressing cells (10^4.59^ TCID_50_/ml) were significantly lower than those in the NC-overexpressing cells (10^5.57^ TCID_50_/ml) after 72 h post infection, whereas the viral titers were not significantly changed in the miR-10a-5p inhibitor-overexpressing cells compared with the NC-overexpressing cells (Figure 2C). Hereafter, we infected the miR-10a-5p mimic-treated cells with PHEV, followed by western blotting and qRT-PCR assays, to detect the intracellular viral proliferation. Cytotoxicity experiment showed that cell viability was not significantly influenced after transfection (Figure 2D). As shown in Figures 2E,F, we observed that the enhanced miR-10a-5p significantly restricts PHEV replication at 72 h post infection. The data suggested the host miR-10a-5p’s widespread negative regulation of PHEV replication and that any antiviral effects at the cellular level should be considered in the context of the overall effect of PHEV-induced miR-10a-5p alteration in the infected hosts.

### Syndecan 1 Is a Target Gene of MiR-10a-5p

**FIGURE 3:**
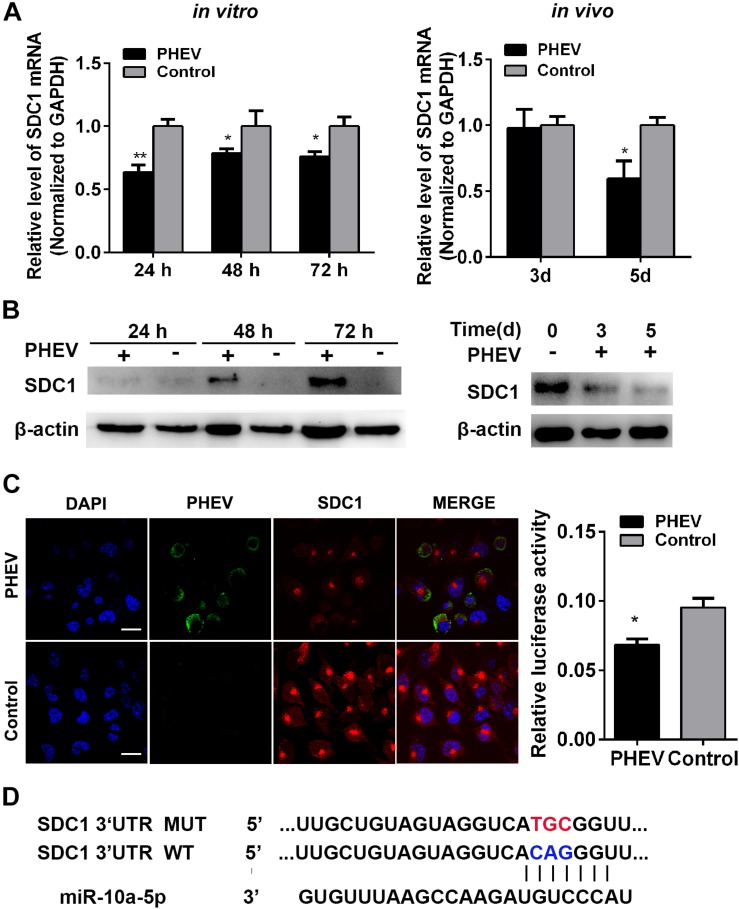
Syndecan 1 (SDC1) expression was inhibited during porcine hemagglutinating encephalomyelitis virus (PHEV) infection. **(A)** N2a cells were incubated with PHEV for 24∼72 h *in vitro*, whereas the BALB/c mice were infected with PHEV for 3 or 5 days *in vivo*. *N* = 4 mice per group, three independent experiments. The endogenous SDC1 mRNA expression was determined by qRT-PCR. **(B)** The lysates from cells or mice that treated as described as (A) were harvested and detected by western blotting using primary antibodies against SDC1 or beta actin. **(C)** N2a cells were incubated with PHEV for 24 h and then fixed and labeled with antibodies against PHEV (green) and SDC1 (red). The cell nucleus was stained with DAPI (blue). Representative micrograph is shown, and quantitative analyses of the Rab3a staining are listed on the right. Bars indicate 20 μm. All of the data are representative of at least three independent experiments (*p < 0.05, **p < 0.01). **(D)** Sequence alignment of miR-10a-5p in TargetScan website and its target site in 3′ UTR of SDC1 mRNA are shown. Model of wild- and mutant-type constructs of SDC1 mRNA 3′ UTR. The red letters indicate the point mutation.

To identify miR-10a-5p target genes, some publically available miRNA target-prediction websites were used, including Pictar, TargetScan, and miRanda. Among all predicted target genes, the potential target Syndecan 1 (SDC1) acts as an important factor that participates in cancer, inflammatory diseases, and pathogen infection (Teng et al., 2012). Its expression was dramatically inhibited during PHEV infection *in vivo* and *in vitro*, which was determined by qRT-PCR and western blotting (Figures 3A,B). An immunofluorescence assay (IFA) analysis further confirmed that SDC1 expression decreased in the PHEV-infected N2a cells compared with the uninfected ones (Figure 3C). Moreover, the sequences of miR-10a-5p and its target site in the 3′-UTR of SDC1 mRNA were then obtained from TargetScan software. It suggests that miR-10a-5p specifically binds to SDC1 mRNA 3′-UTR, through bioinformatics prediction (Figure 3D). Therefore, we hypothesized that SDC1 mRNA was a putative miR-10a-5p target gene and may be involved in PHEV infection.

**FIGURE 4:**
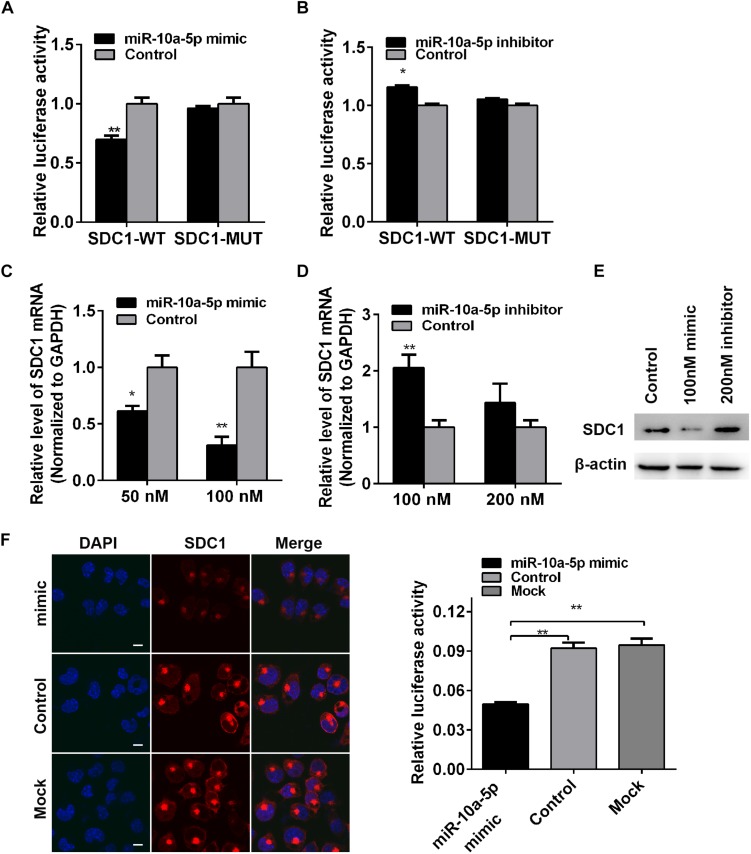
Syndecan 1 (SDC1) mRNA is a target of miR-10a-5p. **(A)** Wild-type (WT) or mutant-type (MUT) reporter constructs of SDC1 3’-UTR were co-transfected with 100 nM of miR-10a-5p mimic into HEK293T cells. After 24 h, the Renilla and firefly luciferase activities were assayed. **(B)** SDC1-WT or SDC1-MUT reporter was co-transfected with 200 nM of miR-10a-5p inhibitor into HEK293T cells for 24 h and were then harvested and subjected to luciferase activities detection. **(C)** N2a cells were transfected with different concentrations of miR-10a-5p mimic for 48 h, and the SDC1 mRNA expression was determined by qRT-PCR. **(D)** N2a cells pre-transfected with miR-10a-5p inhibitor for 48 h were subjected to SDC1 level determination by qRT-PCR. **(E)** Cells were pre-treated as indicated in panels **(C,D)**, lysed, and detected by western blotting assay to examine the level of PHEV protein. Data were normalized to beta actin. All of the data are representative of at least three independent experiments (*p < 0.05, **p < 0.01). **(F)** Representative micrograph of IFA showed the expressed level of SDC1 (red) in the miR-10a-5p mimic- or mock-transfected N2a cells. Nucleus was stained with DAPI (blue). Quantitative analyses of the SDC1 staining are listed on the right. Bars, 20 μm.

To determine whether miR-10a-5p binding to SDC1 depends on this site in the 3′-UTR, we constructed dual-luciferase reporter plasmid carrying the complementary sequence (SDC1-WT) or three base pair mutations (SDC1-MUT) of the seed region in the SDC1 mRNA 3′-UTR (Figure 3D). We found that the luciferase activity of SDC1-WT was significantly decreased when it was co-transfected with the miR-10a-5p mimic for 24 h in the HEK293T cells (Figure 4A). In contrast, miR-10a-5p inhibitors increased the luciferase activity of SDC1-WT reporter (Figure 4B). No significant changes of luciferase activity were observed when co-transfecting with SDC1-MUT reporter and either miR-10a-5p mimics or inhibitors (Figures 4A,B). To further validate the effect of the interaction between miR-10a-5p and SDC1, expression of endogenous SDC1 was analyzed in the N2a cells that pre-transfected with miR-10a-5p mimics or inhibitors. The application of miR-10a-5p mimics significantly decreased the mRNA and protein level of SDC1 at both doses of 50 and 100 nM (Figures 4C,E,F). The promotion effects on SDC1 expression were observed in the miR-10a-5p inhibitor-transfected group (Figures 4D,F). Together, PHEV-induced miR-10a-5p up-regulation was shown to lead to decreased SDC1 protein level, and SDC1 mRNA is a direct target of miR-10a-5p.

### Effects of Syndecan 1 Knockdown on Porcine Hemagglutinating Encephalomyelitis Virus Replication in N2a Cells

**FIGURE 5:**
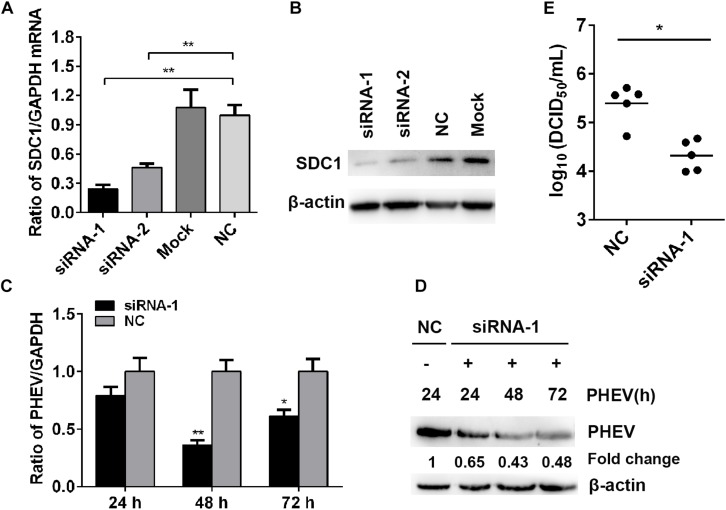
Effects of Syndecan 1 (SDC1) defect in porcine hemagglutinating encephalomyelitis virus (PHEV) replication *in vitro*. **(A)** N2a cells were transfected with 50 nM of SDC1-siRNA-1, SDC1-siRNA-2, or negative control (NC) siRNA, and the expression of SDC1 mRNA was determined by qRT-PCR at 24 h post transfection. **(B)** The protein levels of SDC1 in the cell treated as described in panel **(A)** were determined by western blotting. **(C)** N2a cells were infected with PHEV for 24∼72 h, following SDC1-siRNA-1 transfection, and the levels of the viral genome RNA were detected by qRT-PCR. **(D)** Cells treated as indicated in panel **(C)** were harvested and determined by western blotting. **(E)** Viral titers in the supernatants were measured by TCID_50_/ml assay. Data are shown as means ± SD of at least three independent experiments. *p < 0.05; **p < 0.001.

To confirm whether SDC1 is involved in PHEV infection, we silenced SDC1 with specific siRNA and verified the effects on PHEV replication. The knockdown of gene level was determined by qRT-PCR and western blotting, and we found that the SDC1 mRNA was cut down by more than 70% at 50 nM of siRNA-1-transfected cells (Figures 5A,B). We next determined the effect of SDC1 defect on PHEV infection. N2a cells were transfected with 50 nM of SDC1 siRNA-1, followed by infection of PHEV at 37°C for indicated times. At 24, 48, and 72 h post infection, the levels of PHEV replication were analyzed by qRT-PCR and western blotting, and the viral titers were measured by TCID_50_/ml on 96-well plates as well. We found that the viral load was markedly reduced in these cells (Figures 5C–E). These results demonstrated that SDC1 is positively related with PHEV replication. Therefore, we draw a conclusion by summarizing several tentative findings of the study above and pointing out that the host cells make use of miR-10a-5p to reduce SDC1 levels, in response to PHEV infection.

### MiR-10a-5p Agomir Reduced Porcine Hemagglutinating Encephalomyelitis Virus Replication in Mice

**FIGURE 6:**
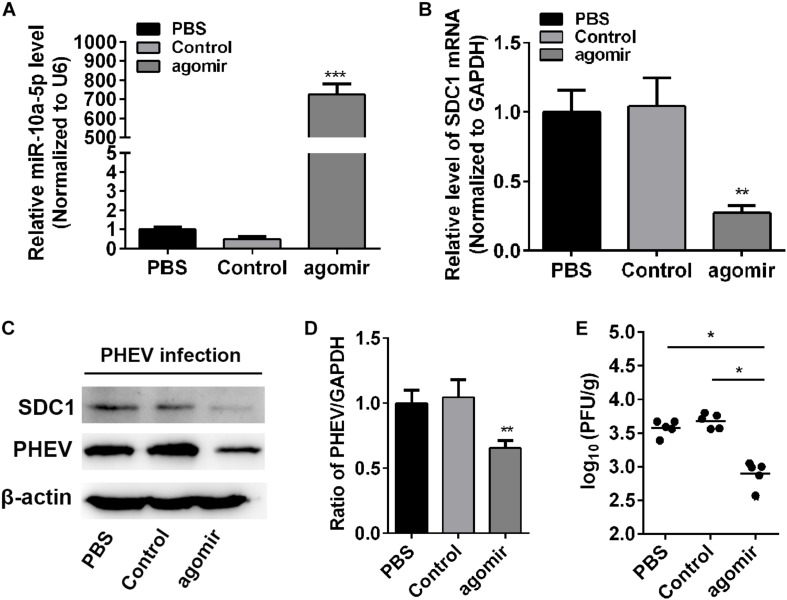
MiR-10a-5p agomir reduced porcine hemagglutinating encephalomyelitis virus (PHEV) replication *in vivo*. BALB/c mice were injected with miR-10a-5p agomir at 1 and 3 days post infection following PHEV infection. *N* = 6 mice per group, three independent experiments. The brain tissues were collected, and qRT-PCR and western blotting assay were performed to assess miR-10a-5p miRNA level **(A)**, Syndecan 1 (SDC1) mRNA transcription **(B)**, SDC1 protein expression **(C)**, and viral replication and load **(C,D)**. Phosphate-buffered saline (PBS)-injected mice were identified as mock injection, whereas mice in the control group were only infected with PHEV without agomir or PBS injection. **(E)** The titer of infectious virus in brain were measured by using a standard plaque assay in N2a cells. Data are shown as means ± SD of at least three independent experiments. *p < 0.05; **p < 0.01; ***p < 0.001.

To determine whether miR-10a-5p was effective against PHEV *in vivo*, the healthy BALB/c mice received an intracerebral injection of miR-10a-5p agomir, a specially chemically modified miRNA agonist that functions by mimicking the entry of endogenous miRNAs into the miRISC complex to regulate the expression of target gene mRNA. PBS-injected mice were used as a mock control. Then, the brain tissues were collected and assigned to assess the expression of miR-10a-5p and SDC1. In the miR-10a-5p agomir-injected mice, an apparent accumulation of miR-10a-5p RNA was observed at 24 h after injection (Figure 6A), thereby leading to SDC1 mRNA and protein level restriction, in compared with the control groups (Figures 6B,C). Next, we infected the mice with PHEV, followed by miR-10a-5p agomir injection at 24 and 72 h post infection. At 5 days post infection, the expression of PHEV was analyzed by qRT-PCR and western blotting. We found that the viral replication appears to be considerably reduced in the brain of PHEV-infected mice after being injected with miR-10a-5p agomir (Figures 6C,D). And also, the brain tissue was weighed and lysed in serum-free MEM, and the viral titration was performed by using a standard plaque assay. As shown in Figure 6E, the infectious viral titer was generally lower in the agomir-treated mice than that in the PBS or control group (Figure 6E). Taken together with the research and analysis above, we concluded that SDC1 is a potential antiviral target, which is controlled by host miR-10a-5p miRNA at the post-transcriptional level in PHEV-induced disease.

## Discussion

Over the past decades, miRNAs have been reported to play critical roles in a variety of cellular processes, including inflammation, cancer, and virus infections (Lee and Dutta, 2009). Of particular note is that many of novel miRNAs and related target genes have been identified to be involved in different stages of virus infections, but in-depth molecular mechanism studies are lacking. Cellular miRNAs can affect virus replication in a direct or indirect way. The former acts as an effected pathway by combining with the virus or viral components. For instance, cellular miR-17 promotes the stability and translation of viral RNA by targeting the 3′-UTR of RNA viruses (Scheel et al., 2016). The latter affects virus replication by targeting host cellular mRNAs (Balasubramaniam et al., 2018). For example, miR-200b and miR-429 suppress the expression of zinc finger E-box binding homeobox 1 (ZEB1) and zinc finger E-box binding homeobox 2 (ZEB2), the two cellular proteins that have key roles to establish and maintain latent infections of Epstein–Barr virus, thus leading to lytic reactivation of latently infected cells (Ellis-Connell et al., 2010). The miRNA-mediated activity plays a critical role in cellular responses to viral infections.

Previous efforts have suggested that the miRNAs mediated variable functions in the infection of PHEV. For example, miR-21a-5p accelerates PHEV proliferation by down-regulating Caskin1 (Lv et al., 2017), and miR-142-5p disrupted the neuronal morphogenesis during PHEV infection by targeting Ulk1 (Li et al., 2017). Yet more miRNA–PHEV interactome research is lacking. Our paper focuses on the host miR-10a-5p and how it evolved to use target gene to join PHEV replication and pathogenesis. The findings presented here firstly identified SDC1 as a promising and novel target of the miR-10a-5p and also provided the evidence that miR-10a-5p could weaken the function of SDC1, which is a cell surface proteoglycan that bears both heparan sulfate and chondroitin sulfate and necessary for host defense mechanisms, angiogenesis, cancer proliferation and invasion, and microbial attachment and entry (Teng et al., 2012).

The therapeutic miRNAs have emerged as novel or functionally relevant targets against the viral infection. Agomir mixture of hsa-mir-127-3p, hsa-mir-486-5p, hsa-mir-593-5p, mmu-mir-487b-5p, and hsa-miR-1-3p protected mice from influenza (H1N1) virus infection (Peng et al., 2018), whereas miR-m01-3-5p and miR-M23-1-5p agomirs have protective effects on reactivation of murine cytomegalovirus (MCMV) in animals (Deng et al., 2017). Therefore, understanding the host regulatory machinery hijacked by miRNAs during PHEV infection has a potential sense in antiviral therapies. Advancement of knowledge on the interaction between RNA viruses and cellular miRNAs focuses on how the RNA viruses have evolved to use host miRNAs to evade antiviral immune responses, leading to enhanced viral replication and pathogenesis. In our study, miR-10a-5p agomir treatment decreased the virus load in the brain tissue of PHEV-infected mice, by miRNA-mediated changes in antiviral-associated effector activity, further supporting the validation of specific miRNAs as potential antiviral agents in the PHEV-induced disease. However, the role of SDC1 activity during PHEV infection remains unclear. Although the data of SDC1 knockdown assay preliminarily suggests that SDC1 might identify as a novel protein involved in the life cycle of PHEV, or specific functional contribution to viral pathogenesis, further experiments would be required to elucidate this point. It is likely that future work will obtain insights into the downstream signaling processes of the SDC1-dependent PHEV replication restriction. And identifying more miRNAs that are involved in down-regulating the innate immune response during PHEV infection is also of great significance.

## Data Availability Statement

The raw data supporting the conclusions of this article will be made available by the authors, without undue reservation to any qualified researcher.

## Ethics Statement

The animal study was reviewed and approved by the Animal Welfare and Research Ethics Committee of the College of Veterinary Medicine, Jilin University, China.

## Author Contributions

SH and WH designed and supervised the experiments. SH, ZL, and YL performed most of the experiments and interpreted the data. JG cultured cells and conducted the statistical analysis. KZ and DC carried out the bioinformatics prediction analysis and prepared the figures. FG and YG drafted the manuscript and GF and WH revised it critically for important intellectual content.

## Conflict of Interest

DC, FG, and YG were employed by the company Yebio Bioengineering Co., Ltd. of Qingdao.

The remaining authors declare that the research was conducted in the absence of any commercial or financial relationships that could be construed as a potential conflict of interest.

## Footnotes

1 http://www.microrna.gr/microT-CDS

2 http://www.microrna.org/microrna/microrna/getDownloads.do

3 https://pictar.mdc-berlin.de/

4 http://www.targetscan.org/mmu_61/

## Supplementary Material

DATA SHEET S1 A miRNA array was previously performed to identify the miRNAs involved in neurological dysfunction as a result of PHEV infection. Supplemental table includes a sheet showing the differentially miRNAs expression profiling with statistical significance among the cerebral cortex samples of mice, which treated with PHEV for 0 days (C1), 3 days (T1), 5 days (T2), respectively.
